# Prevalence and risk factors of lung nodules in a non-smoking Chinese population: a prospective study of low-dose computed tomography screening

**DOI:** 10.1186/s12890-026-04108-2

**Published:** 2026-03-04

**Authors:** Wei Tang, Yanyan Tang, Yi Teng, Jianwei Wang, Lina Zhou, Haohua Zhu, Shijun Zhao, Zewei Zhang, Zhijian Xu, Kai Zhang, Yao Huang, Ning Wu

**Affiliations:** 1 Department of Diagnostic Radiology, National Cancer Center/National Clinical Research Center for Cancer/Cancer Hospital, Chinese Academy of Medical Sciences and Peking Union Medical College, Beijing, 100021 China; 2 Office of Cancer Screening, National Cancer Center/National Clinical Research Center for Cancer/Cancer Hospital, Chinese Academy of Medical Sciences and Peking Union Medical College, Beijing, 100021 China; 3 Department of Medical Oncology, Beijing Key Laboratory of Clinical Study on Anticancer Molecular Targeted Drugs, National Cancer Center/National Clinical Research Center for Cancer/Cancer Hospital, Chinese Academy of Medical Sciences and Peking Union Medical College, Beijing, 100021 China; 4 Department of Nuclear Medicine (PET-CT Center), National Cancer Center/National Clinical Research Center for Cancer/Cancer Hospital, Chinese Academy of Medical Sciences and Peking Union Medical College, Beijing, 100021 China; 5 Department of Cancer Prevention, National Cancer Center/National Clinical Research Center for Cancer/Cancer Hospital, Chinese Academy of Medical Sciences and Peking Union Medical College, Beijing, 100021 China

**Keywords:** Low-dose computed tomography, Non-smoking population, Lung nodule, Women, Second-hand smoke

## Abstract

**Background:**

Low-dose computed tomography (LDCT) has significantly improved early detection of lung cancer, especially among high-risk populations. However, the risk factors and lung nodule distributions in non-smoking populations remain underexplored, particularly in Asia. Therefore, in this study, we aimed to explore the risk factors and delineate the detection rate, including lung nodule distributions discovered using LDCT, in a non-smoking Chinese population.

**Methods:**

This prospective, single-center study included asymptomatic adults who underwent LDCT screening at the National Cancer Center of China between January 2006 and December 2023. Lung nodules were defined as at least one non-calcified nodule, while clinically relevant lung nodules were defined as at least one solid or partially solid nodule, or at least one non-solid nodule. Multivariate logistic regression models were employed to identify risk factors associated with lung nodules. The outcomes included detection rates and distribution of both nodule types.

**Results:**

Of 23,271 participants, lung nodules were detected in 40.1% (9,342/23,271); 4.4% (1,023/23,271) had clinically relevant lung nodules. Risk factors for lung nodule development included female sex (odds ratio [OR] 1.12, 95% confidence interval [CI] 1.06–1.19), SHS exposure (OR 1.59, 95% CI 1.49–1.70), and emphysema (OR 1.49, 95% CI 1.24–1.78). The incidence of lung nodules increased with age, peaking at 70–74 years (OR 3.10, 95% CI 2.53–3.79). Risk factors for clinically relevant lung nodules included increasing age, SHS exposure (OR 1.44, 95% CI 1.22–1.69), and emphysema (OR 1.84, 95% CI 1.36–2.49). Detection rates for both nodule types were positively correlated with age (lung nodules: women 33.7–61.4%, men 32.3–57.5%; clinically relevant lung nodules: women 2.4–12.4%, men 2.2–15.1%).

**Conclusions:**

This real-world study of a non-smoking Chinese population revealed high lung nodule detection rates, with women exhibiting a higher detection rate than men. SHS has emerged as a significant risk factor for both lung and clinically relevant nodules. These findings highlight the importance of refining LDCT screening strategies and risk models for non-smoking populations in Asia.

**Supplementary Information:**

The online version contains supplementary material available at 10.1186/s12890-026-04108-2.

## Background

In 2022, lung cancer exhibited the highest incidence and mortality rates among malignant tumors in men and women in China, surpassing breast cancer as the most prevalent cancer among women [1] .Randomized clinical trials have demonstrated that low-dose computed tomography (LDCT) screening for lung cancer in high-risk groups can significantly reduce mortality [2, 3]. Additionally, opportunistic screening has been associated with lower rates of lung cancer-related deaths and overall mortality [4]. Consequently, LDCT has gained substantial global support from researchers and clinical studies for lung cancer screening (LCS).

The increasing utilization of LDCT has led to a higher detection rate of lung nodules. Over 95% of nodules detected through LDCT are benign [5], presenting a significant challenge in identifying risk factors for these nodules during LCS. Most guidelines rely on nodule density and size for risk assessment. However, recent LCS studies emphasize the need for a comprehensive evaluation of lung nodules, incorporating individual characteristics and nodule-specific attributes into risk grading. While current guidelines and risk prediction models—such as those from the Mayo Clinic and Brock University—incorporate predictors like age and nodule characteristics [6, 7], they are predominantly derived from Western cohorts with heavy smoking histories [8]. These models may not be fully applicable to Asian populations, where non-smokers have historically been underrepresented in risk assessments [9]. Although risk assessments have been conducted in Asian populations, non-smoking participants were not analyzed separately [6]. Furthermore, limited data exists on lung nodule risk factors and the effectiveness of LDCT in detecting nodules among non-smokers in Asian countries, particularly China.

Globally, the lung cancer rate among non-smokers is increasing and is now the seventh most common cause of cancer-related deaths worldwide [7]. Crucially, the epidemiology of lung cancer in Asia differs markedly from that in Western populations, presenting a unique context for screening. While lung cancer in non-smokers is relatively rare in Europe and North America [8], it constitutes a significant and rising public health burden in Asia, particularly among women [9]. This disparity is driven by a distinct interplay of etiology not typically seen in Western cohorts, including genetic susceptibility (such as a higher frequency of EGFR mutations) and specific environmental exposures like indoor air pollution from cooking oil fumes and solid fuel use [10]. Consequently, risk stratification models derived primarily from heavy smokers in Western countries may fail to accurately capture the risk profile of the Asian non-smoking population, necessitating dedicated research to refine local screening strategies [11]. In Asia, lung nodules exhibit unique characteristics, and factors other than tobacco play a significant role in their development. The prevalence of sarcoidosis and other infectious diseases that can influence lung nodules must be considered. While nodules in these conditions are predominantly benign, they often exhibit clinical presentations and imaging features that resemble those of malignant nodules [12, 13]. Consequently, it is clinically essential to study the risk factors, detection rates, and distribution of lung nodules in non-smokers in Asia. Lung cancer is the leading cause of cancer-related deaths in China, and the disease burden among non-smokers is increasing [3]. Furthermore, lung cancer is not uncommon among non-smokers in China. A previous study of ours included 30,468 asymptomatic participants who underwent LDCT screening at our hospital. Of these participants, 21,426 were non-smokers and 9,042 were smokers. The results showed that the overall lung cancer detection rate was 0.9%, with rates of 0.8% and 1.0% among smokers and non-smokers, respectively [4]. Therefore, identifying risk factors specific to this demographic is urgent for refining national health policies and reducing mortality.

Despite these advances, a critical research gap remains. Most existing risk assessment models are derived from Caucasian populations with heavy smoking histories [14], and their applicability to Asian non-smokers—who possess distinct genetic and environmental risk profiles—is questionable. Furthermore, prior studies in Asia have often failed to analyze non-smokers as a separate demographic or have limited their scope to solid nodules only, neglecting the significant burden of sub-solid nodules in this population [6]. Consequently, there is a paucity of large-scale, real-world evidence regarding the specific risk factors and distribution patterns of lung nodules in asymptomatic Chinese non-smokers, impeding the development of tailored screening strategie.

In this prospective, single-center study, we aimed to evaluate the risk, detection rate, and distribution of lung nodules identified using LDCT in a large, real-world cohort of non-smokers in China, utilizing multivariate logistic regression to identify independent predictors. The null hypothesis of this study was that there are no significant associations between the investigated demographic, lifestyle, or clinical factors (specifically age, sex, second-hand smoke exposure, education level, and comorbidities) and the prevalence of lung nodules or clinically relevant lung nodules in the non-smoking Chinese population.

## Methods

### Study design and participants

This prospective, single-center, hospital-based study was approved by the Ethics Committee of the National Cancer Center of China (NCC) /Cancer Hospital of the Chinese Academy of Medical Sciences (Approval No. 14–115/905) and performed in accordance with the principles of the Declaration of Helsinki. All participants provided informed consent, allowing their respective institutions to offer financial support for LCS. This manuscript was written following the STROBE Statement checklist.

This study enrolled asymptomatic individuals who underwent LDCT LCS at the Department of Cancer Prevention, NCC/Cancer Hospital of the Chinese Academy of Medical Sciences, between January 2006 and December 2023. We excluded individuals unable to comprehend the study’s objectives, risks, or benefits, or those who could not provide informed consent. Further, individuals ineligible for curative lung cancer surgery owing to severe heart disease, advanced respiratory disease, or other significant comorbidities, as well as those with a prior diagnosis of lung cancer, were also excluded. Participants completed a lung cancer risk assessment questionnaire developed by our organization in conjunction with LDCT screening.

The questionnaire collected data on demographics, comorbidities, second-hand smoke (SHS) exposure, history of occupational exposure to hazardous substances, and family history of lung cancer. Demographic data included age, sex, educational level, frequency of physical activity, and intake of fruits and vegetables. Educational level was categorized as low (primary school or lower), medium (middle/high school), or high (college/university or higher). Comorbidities included chronic bronchitis or bronchiectasis, emphysema, chronic obstructive pulmonary disease (COPD), asthma, tuberculosis, angina pectoris, diabetes mellitus, and hypertension. Non-smoker was strictly defined as an individual who has smoked fewer than 100 cigarettes in their lifetime. Individuals with any history of smoking, including former smokers and sporadic smokers, were excluded from the study. SHS exposure was defined as passive smoking for over 20 years while living with or working with a smoker. Occupational exposure history was defined as exposure to asbestos or soot for over a year. Physical activity levels were categorized as low, medium, or high. Detailed definitions and variable assignments are provided in Additional file 1.

### LDCT scanning, the imaging assessment, and management of lung nodules

Our LDCT scanning protocol and nodule management followed the I-ELCAP program (I-ELCAP Screening Program 2006), [15] which classifies nodule density based on its ability to obscure the lung parenchyma completely. Nodules were categorized as solid nodules (SN), partial solid nodules (PSN), and non-solid nodules (NSN). Non-calcified nodules (NCN) were defined as nodules that did not meet the typical criteria for benign calcified nodules. Detailed information on LDCT parameters, imaging assessments, and nodule management can be found in the Supplementary document [16].

### Definition and description of lung nodules

The presence or absence of nodules was defined according to the National Lung Screening Trial (NLST) guidelines for positive nodules [17]. A nodule was defined as present if LDCT detected at least one NCN with a long diameter of ≥ 4 mm and absent if no nodule was detected or the detected nodule had a long diameter of < 4 mm. Clinically relevant lung nodules were defined according to I-ELCAP guidelines as positive if LDCT screening revealed at least one SN or PSN with a mean diameter ≥ 5 mm, or at least one NSN with a mean diameter ≥ 8 mm [15]. Positive nodules typically required monthly follow-ups as per management recommendations. Each report described up to 10 larger nodules if multiple nodules were detected.

### Statistical analyses

Continuous variables with a normal distribution are expressed as mean ± standard deviation; variables not following a normal distribution are expressed as median with interquartile range (IQR). Data normality was assessed using the Kolmogorov–Smirnov test. For normally distributed data, quantitative variables were analyzed using the independent two-sample t-test; otherwise, the Mann–Whitney U test was applied. Categorical variables are presented as percentages, with frequencies compared using chi-square or Fisher’s exact tests. Binary logistic regression was selected to analyze the association between risk factors and the dichotomous outcomes (nodule presence: yes/no). To control for potential confounding, variables identified as significant in the univariate analysis (*P* < 0.05) or deemed clinically relevant based on prior literature were entered into a multivariate logistic regression model. This adjustment allowed for the calculation of adjusted odds ratios (ORs) and 95% confidence intervals (CIs) to identify independent risk factors. Model performance was assessed for discrimination using the C-statistic (Area Under the Receiver Operating Characteristic Curve, AUC) and for calibration and goodness-of-fit using the Hosmer-Lemeshow test. A Hosmer-Lemeshow P-value > 0.05 was considered to indicate a good fit. A *P* value of < 0.05 was considered statistically significant. Given the exploratory nature of this study and the use of univariate analysis primarily for variable selection, we did not apply the Bonferroni correction for multiple comparisons to avoid inflating Type II errors. Instead, we focused on the estimates and significance levels derived from the multivariate logistic regression model to draw our primary conclusions. Statistical analyses were performed using SPSS (version 25.0; SPSS Inc.) and R (version 3.6.0; R Foundation for Statistical Computing, Vienna, Austria; https://www.R-project.org/).

## Results

### Characteristics of the study population

**Fig. 1:**
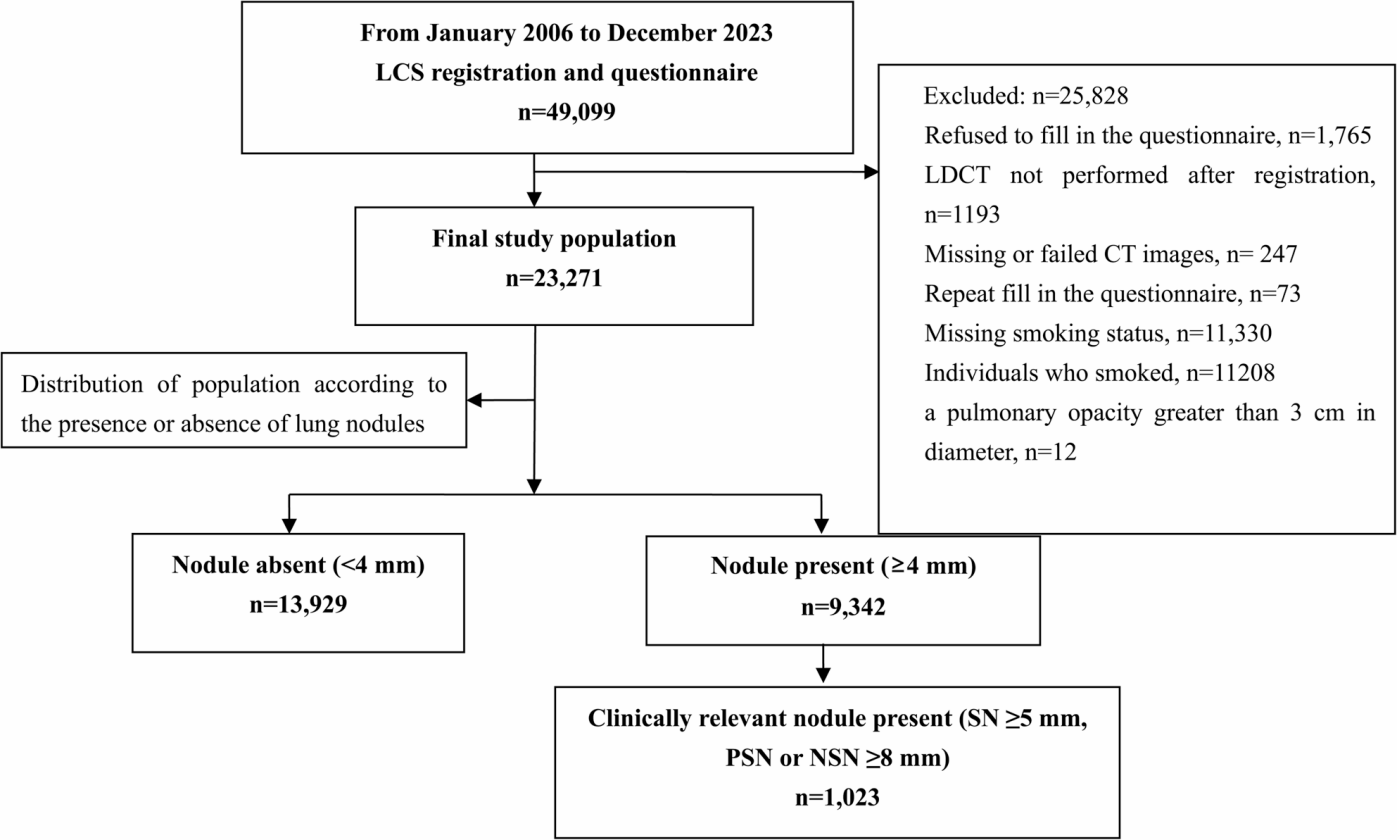
Flowchart of the participant selection and lung nodule presentation based on baseline LDCT screening. CT, computed tomography; LCS, for lung cancer screening; LDCT, low-dose computed tomography; NSN, non-solid nodules; PSN, partial solid nodules; SN, solid nodules

As shown in the flowchart in Fig. 1, 49,099 participants completed the questionnaire between January 2006 and December 2023. A total of 25,828 participants were excluded, including 11,330 with missing smoking status, 11,208 individuals who smoked, 1,765 who refused to fill in the questionnaire, 1,193 who did not undergo LDCT after registration, 247 with missing or failed CT images, 73 duplicate entries, and 12 with a pulmonary opacity greater than 3 cm in diameter. The final study population consisted of 23,271 participants. Based on lung nodule distributions, 59.9% (13,929/23,271) of participants had no nodules, and 40.1% (9,342/23,271) had present nodules.

Table 1 presents the participant stratification based on the presence or absence of lung nodules. The median age of participants with lung nodules was 50 years (IQR: 43–58), which was higher than that of participants without nodules (47 years; IQR: 41–54). The median age of participants with clinically relevant nodules was 54 years (IQR: 47–61), compared with the 48 years (IQR: 41–56) of those without clinically relevant nodules. Participants with lung nodules were more likely to be women and exposed to SHS (all *P* < 0.001). Additionally, hypertension and emphysema were associated with an increased likelihood of lung nodules (all *P* < 0.001). Conversely, increased exercise frequency and higher educational attainment were linked to a reduced likelihood of pulmonary nodules (*P* < 0.05). Among participants with lung nodules, multiple nodules were detected in 42.2% (2,519/5,964) of women and 39.9% (1,346/3,378) of men, with an observed significant difference (*P* = 0.024). Of the clinically relevant nodules, 69.3% (798/1,152) were solid, 20.1% (232/1,152) were partially solid, and 10.6% (122/1,152) were non-solid. In a further analysis of nodule consistency, we observed a distinct sexual dimorphism. Women exhibited a significantly higher proportion of subsolid nodules (SSNs, including part-solid and non-solid nodules) compared to men (36.9% vs. 20.6%, *P* < 0.001). Conversely, solid nodules were more predominant in men (79.4%) than in women (63.1%). This distribution pattern highlights a specific susceptibility to subsolid morphologic changes in the female non-smoking population. The median size of clinically relevant nodules was 6.4 mm (IQR: 5.5–8.8) (Table 2).

**Table 1:** The characteristics of participants stratified according to the presence or absence of lung nodules

Characteristics	Nodules		*P*	Clinically lung nodules		*P*
Total	Present (*n* = 9,342)	Absent (*n* = 13,929)		Present (*n* = 1,023)	Absent (*n* = 22,248)	
Sex			< 0.001			0.600
Male	3,378 (36.2%)	5,493 (39.4%)		382 (37.3%)	8,489 (38.2%)	
Female	5,964 (63.8%)	8,436 (60.6%)		641 (62.7%)	13,759 (61.8%)	
Median (Q1–Q3), years	50 (43–58)	47 (41–54)		54 (47–61)	48 (41–56)	
Age (years) range			< 0.001			< 0.001
< 45	2,725 (29.2%)	5,512 (39.6%)		187 (18.3%)	8,050 (36.2%)	
45–49	1,660 (17.8%)	2,804 (20.1%)		162 (15.8%)	4,302 (19.3%)	
50–54	1,619 (17.3%)	2,204 (15.8%)		176 (17.2%)	3,647 (16.4%)	
55–59	1,314 (14.1%)	1,596(11.5%)		179 (17.5%)	2,731 (12.3%)	
60–64	1,000 (10.7%)	1,007 (7.2%)		129 (12.6%)	1,878 (8.4%)	
65–69	622 (6.7%)	515 (3.7%)		104 (10.2%)	1,033 (4.6%)	
70–74	259 (2.8%)	174 (1.3%)		54 (5.3%)	379 (1.7%)	
≥ 75	143 (1.5%)	117 (0.8%)		32 (3.1%)	228 (1.0%)	
BMI (kg/m^2^)			0.812			0.231
< 18.5	4,685 (50.1%)	6,951 (49.9%)		37 (3.62%)	704 (3.16%)	
18.5–23.9	286 (3.1%)	455 (3.3%)		484 (47.3%)	11,152 (50.1%)	
24–27.9	3,393 (36.3%)	5,043 (36.2%)		380 (37.1%)	8,056 (36.2%)	
≥ 28	978 (10.5%)	1,480 (10.6%)		122 (11.9%)	2,336 (10.5%)	
Educational level			<0.001			<0.001
Low	301 (3.2%)	307 (2.2%)		65 (6.4%)	543 (2.4%)	
Medium	2,650 (28.4%)	3,375 (24.2%)		344 (33.6%)	5,681 (25.5%)	
High	6,391 (68.4%)	10,247 (73.6%)		614 (60.0%)	16,024 (72.0%)	
SHS			<0.001			0.001
No	1,781 (19.1%)	3,596 (25.8%)		194 (19.0%)	5,183 (23.3%)	
Yes	7,561 (80.9%)	10,333 (74.2%)		829 (81.0%)	17,065 (76.7%)	
Family history of lung cancer			0.859			0.507
No	7,858 (84.1%)	11,703 (84.0%)		868 (84.8%)	18,693 (84.0%)	
Yes	1,484 (15.9%)	2,226 (16.0%)		155 (15.2%)	3,555 (16.0%)	
Occupational exposure to hazardous substances			0.480			0.590
No	9,046 (96.8%)	13,463 (96.7%)		986 (96.4%)	21,523 (96.7%)	
Yes	296 (3.2%)	466 (3.3%)		37 (3.6%)	725 (3.3%)	
History of other cancers			0.155			0.127
No	8,826 (94.5%)	13,220 (94.9%)		958 (93.6%)	21,088 (94.8%)	
Yes	516 (5.5%)	709 (5.1%)		65 (96.7%)	1,160 (5.2%)	
COPD			1.000			0.165
No	8,783 (94.0%)	13,096 (94.0%)		951 (93.0%)	20,928 (94.0%)	
Yes	559 (6.0%)	833 (6.0%)		72 (7.0%)	1,320 (6.0%)	
Emphysema			< 0.001			<0.001
No	9,071 (97.1%)	13,700 (98.4%)		971 (94.9%)	21,800 (98.0%)	
Yes	271 (2.9%)	229 (1.6%)		52 (5.1%)	448 (2.0%)	
Angina pectoris			0.562			0.028
No	9,119 (97.6%)	13,614 (97.7%)		989 (96.7%)	21,744 (97.7%)	
Yes	223 (2.4%)	315 (2.3%)		34 (3.3%)	504 (2.3%)	
Diabetes			0.176			0.085
No	8,805 (94.3%)	13,187 (94.7%)		954 (93.3%)	21,038 (94.6%)	
Yes	537 (5.7%)	742 (5.3%)		69 (6.7%)	1,210 (5.4%)	
Hypertension			< 0.001			< 0.001
No	7,509 (80.4%)	11,521 (82.7%)		771 (75.4%)	18,259 (82.1%)	
Yes	1,83 3(19.6%)	2,408 (17.3%)		252 (24.6%)	3,989 (17.9%)	
Physical activity			0.002			< 0.001
Low	118 (1.3%)	125 (0.9%)		23 (2.3%)	220 (1.0%)	
Medium	1,358 (14.5%)	1,885 (13.5%)		168 (16.4%)	3,075 (13.8%)	
High	7,866 (84.2%)	11,919 (85.6%)		832 (81.3%)	18,953 (85.2%)	

*COPD* chronic obstructive pulmonary disease, *SD* standard deviation, *SHS* second-hand smoke, *BMI* body mass index

**Table 2:** Nodules were stratified according to sex

Nodules	Nodule present, *n* (%)		*P* value	Total, *n* (%)	Clinically lung nodules, *n* (%)		*P* value	Total, *n* (%)
Sex	Male, 3,378 (36.2%)	Female, 5,964 (63.8%)		9,342 (100.0%)	Male, 382 (37.3%)	Female, 641 (62.7%)		1,023 (100.0%)
Number			0.024				0.06	
Solitary	2,032 (60.1%)	3,445 (57.8%)		5,477 (58.6%)	338 (88.5%)	58 6(91.4%)		924 (90.3%)
Multiple	1,346 (39.9%)	2,519 (42.2%)		3,865 (41.4%)	44 (11.5%)	55 (8.6%)		99 (9.7%)
Morphology of nodules					437	715	< 0.001	1,152 (100.0%)
Solid	-	-		-	347 (79.4%)	451 (63.1%)		798 (69.3%)
Part-solid	-	-		-	55 (12.6%)	177 (24.8%)		232 (20.1%)
Non-solid	-	-		-	35 (8.0%)	87 (12.1%)		122 (10.6%)
Nodule size, mm	-	-		-			0.095	
Median (Q1–Q3)	-	-		-	6.2 (5.5–8.4)	6.5 (5.5–8.9)		6.4 (5.5–8.8)

*Q* quartile, *SD* standard deviation

*Note*: The values for "Morphology of nodules" (solid, part-solid, and non-solid) represent the total count of nodules detected (*n*=1,152), rather than the number of participants (*n*=1,023), as some individuals presented with multiple nodules of different types

### Risk factors for the presence of nodules

**Fig. 2:**
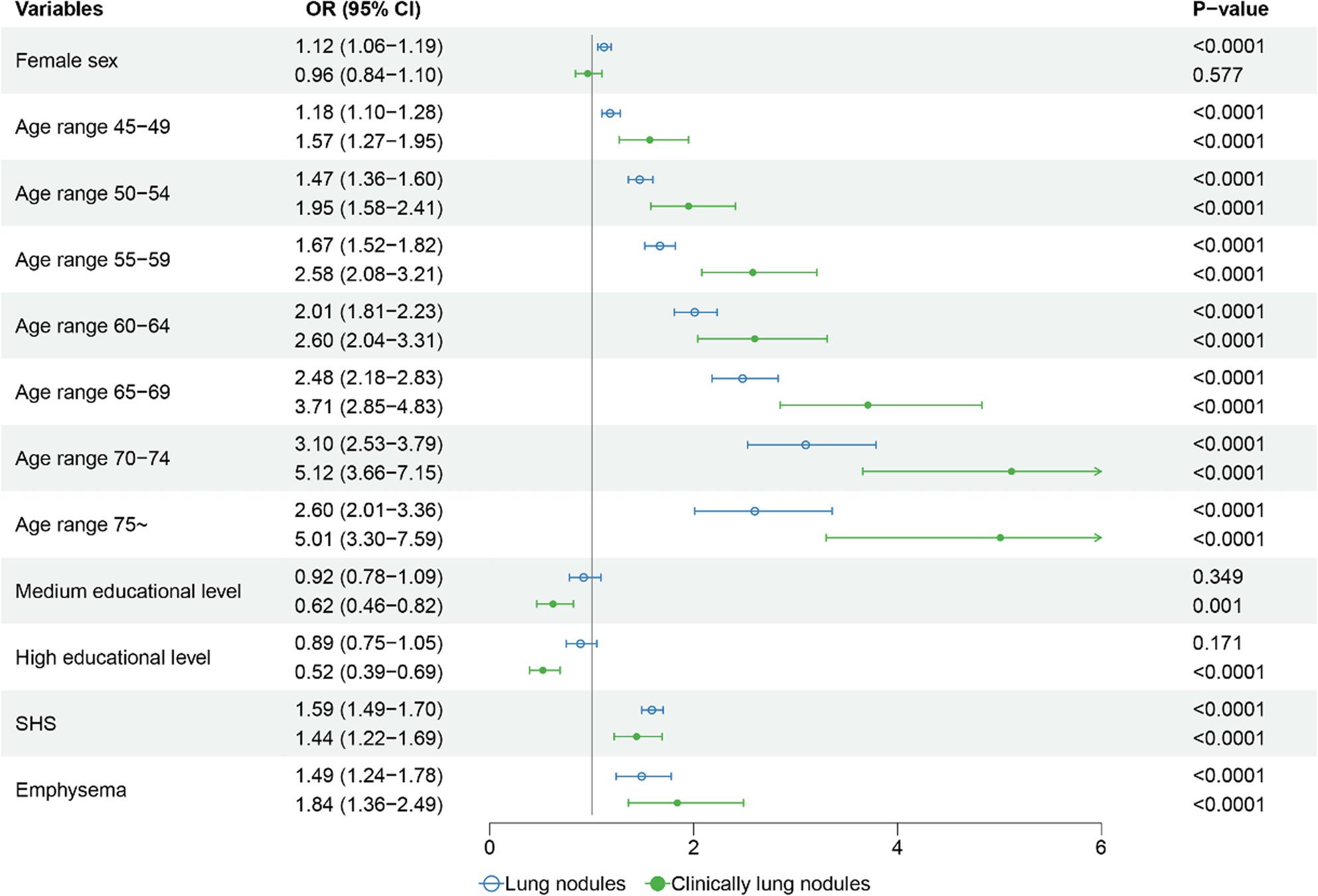
Forest plot of the multifactorial analysis of non-smoking participants with at least one lung nodule. Low-dose computed tomography scans with at least one lung nodule are compared using odds ratios for each factor. Nodule types are classified into two categories: the first being at least one non-calcified nodule with a long diameter greater than or equal to 4 mm; clinically relevant nodules were at least one non-calcified SN or PSN with a mean diameter of ≥5 mm, or at least one NSN with a mean diameter of ≥8 mm. SN, solid nodule; PSN, partial solid nodule; NSN, non-solid nodule; SHS, second-hand smoke; OR, odds ratio; CI, confidence interval

Figure 2 displays a forest plot of the multifactorial analysis of non-smoking participants with at least one lung nodule. Risk factors for different nodule types, as identified by univariate logistic regression, are presented in Additional file 2.Multivariate regression analysis identified female sex as a significant risk factor, associated with a 12% higher odds of lung nodules (OR 1.12, 95% CI 1.06–1.19). Notably, environmental and clinical factors showed more substantial effect sizes: SHS exposure increased the odds by approximately 59% (OR 1.59, 95% CI 1.49–1.70), and emphysema was associated with a 49% increase in risk (OR 1.49, 95% CI 1.24–1.78). Age demonstrated the strongest gradient effect; compared to the youngest group, the risk increased progressively, reaching a three-fold peak in individuals aged 70–74 years (OR 3.10, 95% CI 2.53–3.79).For clinically relevant lung nodules, the impact of comorbidities was even more pronounced. Emphysema nearly doubled the likelihood of detection (OR 1.84, 95% CI 1.36–2.49), while SHS exposure was associated with a 44% increase in odds (OR 1.44, 95% CI 1.22–1.69). Age remained a potent driver, with the risk escalating to a five-fold peak in the 70–74 age group compared to younger individuals (OR 5.12, 95% CI 3.66–7.15). Conversely, higher educational attainment served as a significant protective factor, reducing the odds by nearly half (OR 0.52, 95% CI 0.39–0.69) (Additional file 3). The multivariate model demonstrated adequate discrimination with AUC: 0.6586 (95% CI: 0.6416–0.6757). The Hosmer-Lemeshow test indicated good goodness-of-fit (X^2^ = 3.5219, *P* = 0.8975), suggesting that the model was well-calibrated.

### Lung nodule detection rates stratified by sex and age

To evaluate the distribution of lung nodules by sex and age, we analyzed detection rates in the “nodule present” category. Female participants had a higher overall detection rate than male participants (41.4% [5,964/14,400] vs. 38.1% [3378/8,871], *P* < 0.001). Detection rates were higher in female participants than in male participants in the 50–54, 55–59, and 65–69 age groups, with significant differences (*P* < 0.05). In the category of clinically relevant lung nodules, detection rates were 4.5% (641/14,400) in female participants and 4.3% (382/8,871) in male participants, with no significant difference between groups (Table 3).

**Table 3:** Lung nodule detection rate stratified by sex and age

	Nodule present, % (*n*/*n*)		*P* value	Total, % (*n*/*n*)	Clinically lung nodules, % (*n*/*n*)		*P* value	Total, % (*n*/*n*)
Total	Male, 38.1 (3,378/8,871)	Female, 41.4 (5,964/14,400)	< 0.001	40.1, (9,342/23,271)	Male, 4.3 (382/8,871)	Female, 4.5 (641/14,400)	0.600	4.4, (1,023/23,271)
Age (years) range								
< 45	32.3 (1,154/3,572)	33.7 (1,571/4,665)	0.190	33.1 (2,725/8,237)	2.2 (77/3,572)	2.4 (110/4,665)	0.541	2.3 (187/8,237)
45–49	36.8 (614/1,669)	37.4 (1,046/2,795)	0.671	37.2 (1,660/4,464)	4.0 (66/1,669)	3.4 (96/2,795)	0.369	3.6 (162/4,464)
50–54	39.6 (514/1,298)	43.8 (1,105/2,525)	0.014	42.3 (1,619/3,823)	4.2 (55/1,298)	4.8 (121/2,525)	0.438	4.6 (176/3,823)
55–59	42.4 (430/1,015)	46.6 (884/1,895)	0.027	45.2 (1,314/2,910)	6.0 (61/1,015)	6.2 (118/1,895)	0.816	6.2 (179/2,910)
60–64	48.6 (304/625)	50.4 (696/1,382)	0.475	49.8 (1,000/2,007)	6.2 (39/625)	6.5 (90/1,382)	0.818	6.4 (129/2,007)
65–69	50.5 (208/412)	57.1 (414/725)	0.031	54.7 (622/1,137)	11.2 (46/412)	8.0 (58/725)	0.075	9.1 (104/1,137)
70–74	57.5 (100/174)	61.4 (159/259)	0.415	59.8 (259/433)	12.6 (22/174)	12.4 (32/259)	0.929	12.5 (54/433)
≥ 75	50.9 (54/106)	57.8 (89/154)	0.275	55.0 (143/260)	15.1 (16/106)	10.4 (16/154)	0.256	12.3 (32/260)

## Discussion

### Key findings

This large LCS cohort study, conducted in a hospital in China, examined the risk factors and detection rates of lung nodules, with a focus on nodule detection rates and distribution by sex and age in a non-smoking population. Independent risk factors included female sex, SHS exposure, emphysema, and older age, while higher education levels were associated with lower odds of clinically relevant lung nodules. Among the non-smoking population in China, the detection rates of lung nodules and clinically relevant lung nodules were 40.1% (9,342/23,271) and 4.4% (1,023/23,271), respectively. Women had a significantly higher detection rate of lung nodules (41.4%, 5,964/14,400) than men (38.1%, 3,378/8,871; *P* < 0.001). The detection rate for clinically relevant lung nodules was also slightly higher in women (4.5%, 641/14,400) than in men (4.3%, 382/8,871).

### Strengths and limitations

This study possesses several notable strengths that enhance the validity and significance of its findings. Its primary strength lies in the inclusion of a large, prospective, real-world cohort of over 23,000 asymptomatic individuals, making it one of the most substantial investigations of its kind. A key novelty is its specific focus on a non-smoking Chinese population, a critical demographic that has been significantly underrepresented in previous lung cancer screening research, which has predominantly centered on Caucasian smokers. By concentrating on non-smokers, this study directly addresses a crucial knowledge gap regarding the etiology and prevalence of lung nodules in a population where risk factors beyond tobacco play a significant role. The use of standardized low-dose computed tomography (LDCT) protocols and established nodule definition criteria (I-ELCAP, NLST) ensures methodological rigor and enhances the comparability of our findings with international studies. Furthermore, the collection of comprehensive data.

Current guidelines exclude non-smokers, yet our study shows a high nodule detection rate (40.1%) and a specific burden of clinically relevant nodules in this group. Identifying these risk factors allows for the creation of tailored prediction models (precision risk stratification) to determine which non-smokers warrant screening, moving beyond the “smoking-only” paradigm that misses many Asian lung cancer cases.

Despite these strengths, we acknowledge some limitations. First, the study was conducted at a single center, the National Cancer Center of China. Although this center recruits participants from diverse geographical regions, the findings may not be fully generalizable to the entire Chinese population, and potential selection biases inherent to a hospital-based cohort may exist. Second, this analysis is cross-sectional, focusing exclusively on baseline LDCT screening data. Consequently, we could not assess the longitudinal evolution of nodules—such as growth, resolution, or the incidence of new nodules—nor could we report on the ultimate rates of malignancy. This precludes any conclusions about the long-term clinical significance of the detected nodules. Thirdly, the reliance on a self-administered questionnaire for data on comorbidities and lifestyle exposures, such as second-hand smoke, is subject to recall bias, which may have influenced the risk factor analysis. Specifically, while we defined SHS exposure by duration, the inability to quantify smoke intensity serves as a limitation affecting the precision of our risk estimates. Fourth, regarding the radiologic workflow, although standardized protocols (I-ELCAP) were followed, a formal quantitative agreement evaluation (e.g., Kappa or ICC) was not performed for this large-scale real-world cohort. The lack of specific inter-reader variability data is a limitation of this retrospective analysis. Additionally, potential unmeasured confounders remain a limitation. For instance, while we assessed general environmental risks, we did not explicitly quantify exposure to cooking oil fumes—a significant risk factor for lung abnormalities in Chinese women—or obtain specific outdoor air pollution indices (PM2.5) for participants’ residential areas. Precise quantification of smoke intensity was not fully captured in this real-world cohort. Furthermore, genetic susceptibility markers (such as EGFR mutation status) were not assessed in this screening cohort, which may influence nodule development independent of environmental exposures. The primary drawback of focusing on nodule risk factors is the potential for overmanagement. Since over 95% of LDCT-detected nodules are benign, and our study found a high overall detection rate (40.1%), there is a risk that identifying these individuals could lead to anxiety and unnecessary follow-up for indolent lesions.

### Comparison with similar research

A strong correlation between lung nodules and increasing age has been consistently reported globally in both smoking and non-smoking populations [6, 14, 18–20]. For instance, a study in Western Europe showed that individuals aged ≥ 66 years were more than twice as likely to have lung nodules (≥ 100 mm^3^) compared with those aged 45–55 years [14]. Similarly, our study found that older age groups were more likely to develop lung nodules, with those aged 70–74 years being more than three times as likely as those aged < 45 years (OR 3.10, 95% CI 2.53–3.79). The risk was even higher for clinically relevant nodules, with individuals aged 70–74 years being approximately five times as likely to have lung nodules as those aged < 45 years (OR 5.12, 95% CI 3.66–7.15). Age-related cumulative exposure to risk factors, including asbestos, radioactive materials, and air pollution, likely contributes to this increased risk, particularly in regions such as China, where air pollution is more severe than in the United States [21].

Female sex has emerged as a significant risk factor for lung cancer in Asia. A meta-analysis of 141,396 ever-smokers and 109,251 non-smokers revealed that non-smoking women in Asia had a higher lung cancer risk than non-smoking men, comparable to that of high-risk smokers (≥ 30 pack-years; OR = 0.99, 95% CI: 0.65–1.50) [22]. Our study further established that female sex is an independent risk factor for the development of lung nodules. In contrast, Western populations often consider male sex to be at higher risk for pulmonary nodules, likely due to differences in smoking behaviors. Lung nodule prevalence increases with prolonged and intense smoking, showing a higher incidence among male smokers than among female smokers. In the United States, 90.4% of men and 84.3% of women diagnosed with lung cancer are smokers, while in Europe, the figures are 93.3% for men and 68% for women [23–25]. Genetic factors may also play a role, as evidenced by differences in oncogene alterations between Asians and Caucasians, including epidermal growth factor receptor mutations, occurring in 40–55% of Asians compared to 15–25% in Caucasians [26, 27].

SHS exposure is another well-established risk factor for lung cancer [28]. A meta-analysis of 20 randomized controlled trials in Chinese populations, published between 1996 and 2015, revealed that SHS exposure at work increased lung cancer risk by 1.78 (OR 1.78, 95% CI 1.29–2.44), while SHS exposure at home raised the risk by 1.53 times (OR 1.53, 95% CI 1.01–2.33) [29]. The “2020 China Smoking and Health Hazards Report” published by the National Health Commission of China revealed that up to 68.1% of non-smokers in China are exposed to SHS in public places [30]. Similarly, our study identified an association between SHS exposure and an increased risk of lung nodules.

Higher education levels were associated with a reduced risk of developing clinically relevant lung nodules. The findings align with the results of a European study that reported an increased risk of lung nodules among smokers with lower education levels [14]. Higher education levels have been shown to protect against lung cancer [31]. Education, a proxy for socioeconomic status, may influence exposure to environmental carcinogens and high-risk occupations, affecting lung nodule prevalence.

Emphysema, a known independent risk factor for lung cancer [32, 33], was associated with a three-fold higher incidence density of lung cancer in patients with emphysema than in those without emphysema. Multifactorial analysis showed that the presence of emphysema on LDCT was an independent risk factor for lung cancer, even in the absence of airway obstruction (RR 2.10, 95% CI 0.79–5.58) [32]. Our study also indicates that pulmonary emphysema is an independent risk factor for the development of lung nodules. Reduced lung function in individuals with emphysema may impair the clearance of abnormal cells, thereby increasing the likelihood of lung nodule formation. Moreover, emphysema patients are often more prone to lung infections and other comorbidities, which can occasionally present as lung nodules.

Using the NLST diameter threshold of 4 mm, this study reported a lung nodule detection rate of 40.1% (9,342/23,271), consistent with the findings of a population-based study in Northern Europe (42.0%, 4,377/10,431), which used a comparable threshold [34]. Our study reported a higher lung nodule detection rate using a 4-mm threshold than that reported by other studies employing the same threshold, including a multicenter study in Shanghai (29.9%, 4,336/14,506) and a single-center cross-sectional study in Korea (16.2%, 6,066/37,436) [35, 36]. Although the above studies were conducted on both non-smokers and smokers. However, the detection rate in our study was lower than that in a previous Japanese study (42.6%), which included both smoking and non-smoking individuals and used a 5-mm threshold for positivity [18]. Using the I-ELCAP (2006) definition of a clinically relevant nodule —solid or partially solid NCN ≥ 5 mm or non-solid non-calcified lung nodules ≥ 8 mm—the detection rate in our cohort was 4.4% (1,023/23,271). Additionally, our study revealed a higher detection rate of lung nodules in women than in men when applying the 4-mm threshold (42.2% vs. 38.1%, respectively), with multiple nodules also being more common in women than in men (42.2% vs. 39.9%). In contrast, a Nordic population-based study found higher rates of lung nodules and multiple nodules in men across all age groups, except in the 70–74.9-year-old and ≥ 80-year-old subgroups [14]. Conversely, this study, conducted in a predominantly non-smoking cohort, included only SN. The observed discrepancies may be attributed to differences in inclusion criteria, population characteristics, imaging protocols, and interpretation methods. To provide a clear quantitative synthesis of how our findings in Chinese non-smokers compare with other major screening cohorts, we present a summary in Table 4.

**Table 4:** Comparison of lung nodule prevalence and risk factors across major population-based studies

Study (Region)	Population & Smoking Status	Nodule Definition (Threshold)	Prevalence	Key Risk Factors Identified
Current Study (China)	Never-smokers only (*N* = 23,271)	≥ 4 mm	40.1% (Overall)	Female sex, Age, SHS, Emphysema
Cai et al. [14] (Netherlands)	General population (Mixed); 39.9% Never-smokers	Volume ≥ 30mm^3^ (≈ 4 mm)	41.8% (Overall) 38.8% (Never-smokers)	Male sex, Older age, Smoking, Asbestos, COPD
He et al. [6] (North China)	General population (Mixed); 24% Smokers	All detected nodules (mostly < 5 mm)	26.3% (Overall)	Smoking, Dust/Pesticide exposure, History of lung disease
Kakinuma et al. [18] (Japan)	Screening cohort (Mixed); 49.7% Never-smokers	≥ 5 mm	42.6% (Overall)	

*Abbreviations: SHS* Second-hand smoke, *COPD* Chronic Obstructive Pulmonary Disease

To the best of our knowledge, no prior study of this scale has comprehensively evaluated risk factors, detection rates, and distribution patterns of lung nodules in non-smoking Asian populations. Most previous studies have focused on high-risk populations with a history of smoking or on Caucasian cohorts [6, 14]. Our study utilized LDCT screening in asymptomatic non-smokers, collecting extensive epidemiological and imaging data. These findings provide a critical basis for understanding lung nodule characteristics in Asian non-smokers and align with current trends in risk stratification and nodule evaluation using LDCT LCS.

### Explanations of findings

The high detection rate of lung nodules (40.1%) and clinically relevant nodules (4.4%) in this large cohort of 23,271 asymptomatic non-smokers underscores the significant burden of pulmonary abnormalities in this population. Our multifactorial analysis provides explanations for this prevalence by identifying several independent risk factors. The strong positive correlation between nodule prevalence and advancing age, peaking in the 70–74 age group, likely reflects biological aging processes. These include the cumulative DNA damage from long-term exposure to environmental carcinogens (such as particulate matter and SHS) and the age-related decline in pulmonary immune surveillance, which impairs the clearance of abnormal cellular proliferation.

Furthermore, the findings establish female sex and exposure to SHS as significant risk factors. The increased risk for women aligns with emerging evidence of higher susceptibility to lung carcinogens in non-smoking Asian females, potentially due to genetic or hormonal factors. Asian women who have never smoked exhibit a unique genetic profile compared to Western populations or smokers. Lung cancer in this demographic is predominantly adenocarcinoma, which is strongly associated with oncogenic driver mutations. Research indicates that EGFR mutations occur in a substantial proportion of Asian female lung cancer patients (up to 50–70%), a rate significantly higher than in Caucasian populations. Furthermore, specific genetic polymorphisms, such as those in CYP1A1 (cytochrome P450 1A1), may alter the metabolic activation of carcinogens, making women more susceptible to DNA damage from environmental exposures like cooking fumes or second-hand smoke [37]. Endogenous hormonal factors likely play a significant role in lung carcinogenesis. Estrogen receptors are expressed in lung tissue and can promote cell proliferation [38]. There is evidence of cross-talk between estrogen signaling pathways and the EGFR pathway, where estrogen may upregulate EGFR expression or activation, potentially accelerating nodule growth and malignant transformation in women [39].

The notable risk associated with SHS exposure (OR 1.59, 95% CI 1.49–1.70) highlights the profound impact of environmental tobacco smoke as a pulmonary irritant and carcinogen. Similarly, the link between emphysema and an elevated risk for both general and clinically relevant nodules is consistent with its known role in impairing lung clearance mechanisms and creating a chronic inflammatory state conducive to nodule formation.

### Implications and actions needed

The results of this study have significant implications for both public health initiatives and clinical lung cancer screening (LCS) strategies in China. The data compellingly suggest that current screening paradigms, which are heavily weighted toward smoking history, may fail to identify a large segment of the at-risk population. The high nodule prevalence among non-smokers, particularly in women, older adults, and those with SHS exposure or emphysema, indicates that these factors are critical for risk stratification.

Based on these findings, several actions are warranted. First, from a public health perspective, targeted educational campaigns should be developed to raise awareness among non-smokers about the risks of SHS and other environmental exposures. These efforts should prioritize high-risk demographics identified in our study. Second, and most critically, we propose specific revisions to national screening guidelines to operationalize these findings. Current criteria relying heavily on ‘pack-years’ should be expanded to include a ‘Non-smoking High-Risk’ category. Based on our data, this category should target individuals aged 50–74, particularly women, who have a history of significant SHS exposure (> 20 years) or radiologically confirmed emphysema. Practically, we recommend integrating a brief ‘Risk Stratification Questionnaire’ into community health screenings and respiratory clinics. This tool would flag non-smokers with these specific risk factors for referral to LDCT, thereby transforming an ‘opportunistic’ practice into a systematic policy strategy without overburdening the healthcare system with low-risk screenings.

Our Lung Imaging Reporting and Data System (Lung-RADS) classification results revealed that the majority of participants were categorized as Category 2 (64.6%) [40], a group that typically includes small subsolid nodules (SSNs) common in Asian non-smokers. This distribution underscores the predominantly indolent nature of the detected lesions. However, given that 37.8% of lung cancers in our non-smoking cohort manifested as non-solid nodules [40], distinguishing between indolent precursors and clinically relevant early adenocarcinoma is paramount. Recent studies highlight a significant ‘East-West difference’ in nodule patterns, with Asian populations exhibiting a much higher prevalence of subsolid nodules (SSNs) compared to Western cohorts (12.6% vs. 3.6%) [41]. To balance early detection with the avoidance of overdiagnosis, we support the adoption of an ‘Active Surveillance’ strategy for persistent SSNs, consistent with recent recommendations for Asian populations [42]. Specifically, for Lung-RADS Category 2 SSNs, long-term annual follow-up allows clinicians to monitor for interval growth or the development of solid components—key indicators of invasiveness —thereby preventing unnecessary surgical intervention for potentially non-progressive lesions while ensuring timely treatment for those that demonstrate malignant behavior [43, 44].

Given the unique epidemiological features of lung cancer in Asian populations, particularly the high prevalence among non-smokers and the predominance of subsolid nodules, directly adopting Western screening protocols such as the United States Preventive Services Task Force (USPSTF) guidelines or standard Lung-RADS may be insufficient. Our findings, consistent with recent large-scale studies, underscore the urgent need for population-specific management strategies that emphasize precision risk stratification and individualized surveillance. First, risk assessment models must be tailored to the Asian context. Western guidelines predominantly target heavy smokers, potentially excluding high-risk never-smokers who account for over 40% of lung cancer cases in China. To address this, we advocate for the integration of data-driven prediction tools, such as the China NCC-LCm2021 models, which incorporate non-smoking risk factors to define precise risk thresholds (≥ 0.47% for never-smokers), thereby significantly enhancing screening efficiency [11]. Furthermore, the implementation of advanced triage systems like the C-Lung-RADS, which fuses imaging features with AI-predicted malignancy probabilities and clinical demographics, has shown superior sensitivity (87.1% vs. 63.3%) compared to the standard Lung-RADS v2022 in Chinese cohorts [45].

## Conclusions

Our study demonstrates that older age, female sex, SHS exposure, and emphysema are associated with pulmonary nodules in an asymptomatic, non-smoking Asian population. These findings enhance understanding of LCS in China and support the refinement of screening eligibility criteria. Additionally, our research reveals a high detection rate of lung nodules among the non-smoking Chinese population, offering detailed distribution patterns that provide valuable insights for managing pulmonary nodules in this demographic.

## Supplementary Information

Additional file 1: Variable assignment.

Additional file 2: Risk factors for different types of nodules by univariate logistic regression analysis.

Additional file 3: Risk factors for different types of nodules by multivariate logistic regression analysis.

Additional file 4.

## Data Availability

The datasets used and/or analyzed during the current study are available from the corresponding author on reasonable request.

## Abbreviations

LC: Lung Cancer
LCS: Lung Cancer Screening
LDCT: Low-dose Computed Tomography
NLST: National Lung Screening Trial
SHS: Second-hand Smoke
COPD: Chronic Obstructive Pulmonary Disease
I-ELCAP: International Early Lung Cancer Action Program
SN: Solid Nodules
PSN: Partial Solid Nodules
NSN: Non-solid Nodules
IQR: Interquartile Range
USPSTF: United States Preventive Services Task Force
Lung-RADS: Lung Imaging Reporting and Data System
