# Supplementary material for: Prevalence and risk factors of lung nodules in a non-smoking Chinese population: a prospective study of low-dose computed tomography screening
Source: BMC Pulm Med. 2026 Mar 4;26:165. doi: 10.1186/s12890-026-04108-2 (PMC13067505; doi:10.1186/s12890-026-04108-2)
Supplement: Supplementary file 1 — Additional file 1: Variable assignment. [file 12890_2026_4108_MOESM1_ESM.docx]

**Additional file 1: Variable assignment**

| Variables | Assignment | Definition |
| --- | --- | --- |
| Group 1 | 1: nodule absent | Participants have no lung nodules or detected lung nodules <4 mm in long diameter |
|  | 2: nodule present | Participants have at least one non-calcified nodule ≥4 mm in long diameter detected |
| Group 2 | 1: nodule absent | Participants have no lung nodules or detected SN or PSN with a mean diameter of <5 mm, or NSN with a mean diameter of <8 mm |
|  | 2: nodule present | Participants have at least one SN or PSN with a mean diameter of ≥5 mm, or at least 1 NSN with a mean diameter of ≥8 mm detected |
| Age at CT scan (years) | 1:<45 | Age at the time of having LDCT scan |
|  | 2:45–49 |  |
|  | 3:50–54 |  |
|  | 4:55–59 |  |
|  | 5:60–64 |  |
|  | 6:65–69 |  |
|  | 7:70–74 |  |
|  | 8: ≥75 |  |
| SHS exposure | 0: No; 1: Yes | Defined as passive smoking for more than 20 years by living or working with a smoker |
| BMI (kg/m^2^) | 0: Normality, 18.5–23.9 | Weight (kg) / height in meter squared (m^2^) |
|  | 1: Underweight, <18.5 |  |
|  | 2: Preobesity, 24–27.9 |  |
|  | 3: Obesity, ≥28 |  |
| Educational level | 0: Low | Participant educated to primary level or below |
|  | 1: Medium | Participant educated to junior or senior high school |
|  | 2: High | Participants educated to college/university or higher |
| Physical activity | 0: Low ≤2 sessions/week  1: Medium: 3-5 sessions/week  2: High ≥6 sessions/week | Each exercise session lasted more than 30 min (including ball games, running, park activities, among others) |
| Family history of lung cancer | 0: No; 1: Yes | Based on self-reporting in participant questionnaires |
| Occupational exposure to hazardous substances | 0: No; 1: Yes | Based on self-reporting in participant questionnaires |
| History of other cancers | 0: No; 1: Yes | Based on self-reporting in participant questionnaires |
| COPD | 0: No; 1: Yes | Based on self-reporting in participant questionnaires |
| Asbestosis | 0: No; 1: Yes | Based on self-reporting in participant questionnaires |
| Angina pectoris | 0: No; 1: Yes | Based on self-reporting in participant questionnaires |
| Diabetes | 0: No; 1: Yes | Based on self-reporting in participant questionnaires |
| Hypertension | 0: No; 1: Yes | Based on self-reporting in participant questionnaires |
| Emphysema | 0: No; 1: Yes | Based on self-reporting in participant questionnaires |
| Coronary artery calcification | 0: No; 1: Yes | Based on self-reporting in participant questionnaires |

BMI, body mass index; COPD, chronic obstructive pulmonary disease; CT, computed tomography; LDCT, low-dose computed tomography; NSN, non-solid nodules; PSN, partial solid nodules; SHS, second-hand smoking; SN, solid nodule
