# Supplementary material for: Prevalence and risk factors of lung nodules in a non-smoking Chinese population: a prospective study of low-dose computed tomography screening
Source: BMC Pulm Med. 2026 Mar 4;26:165. doi: 10.1186/s12890-026-04108-2 (PMC13067505; doi:10.1186/s12890-026-04108-2)
Supplement: Supplementary file 2 — Additional file 2: Risk factors for different types of nodules by univariate logistic regression analysis. [file 12890_2026_4108_MOESM2_ESM.docx]

**Additional file 2: Risk factors for different types of nodules by univariate logistic regression analysis**

| **Characteristics** | **Lung noodles** | | **Clinically** **relevant nodules** | |
| --- | --- | --- | --- | --- |
| Sex |  |  |  |  |
| Male | Ref. |  | Ref. |  |
| Female | 1.15 [1.09–1.21] | <0.001 | 1.04 [0.91–1.18] | 0.600 |
| Age (years) range |  |  |  |  |
| <45 | Ref. |  | Ref. |  |
| 45–49 | 1.20 [1.11–1.29] | <0.001 | 1.62 [1.31–2.01] | <0.001 |
| 50–54 | 1.49 [1.37–1.61] | <0.001 | 2.08 [1.68–2.56] | <0.001 |
| 55–59 | 1.67 [1.53–1.82] | <0.001 | 2.82 [2.29–3.48] | <0.001 |
| 60–64 | 2.01 [1.82–2.22] | <0.001 | 2.96 [2.35–3.72] | <0.001 |
| 65–69 | 2.44 [2.15–2.77] | <0.001 | 4.33 [3.38–5.56] | <0.001 |
| 70–74 | 3.01 [2.47–3.67] | <0.001 | 6.13 [4.45–8.45] | <0.001 |
| ≥75 | 2.47 [1.93–3.17] | <0.001 | 6.04 [4.06–8.99] | <0.001 |
| BMI, kg/m^2^ |  |  |  |  |
| 18.5–23.9 | Ref. |  | Ref. |  |
| 18.5 | 0.93 [0.80–1.09] | 0.370 | 1.21 [0.86–1.71] | 0.274 |
| 24–27.9 | 1.00 [0.94–1.06] | 0.952 | 1.09 [0.95–1.25] | 0.235 |
| ≥28 | 0.98 [0.90–1.07] | 0.663 | 1.20 [0.98–1.47] | 0.075 |
| Educational level |  |  |  |  |
| Low | Ref. |  | Ref. |  |
| Medium | 0.80 [0.68–0.95] | 0.009 | 0.51 [0.38–0.67] | ＜0.001 |
| High | 0.64 [0.54–0.75] | <0.001 | 0.32 [0.24–0.42] | ＜0.001 |
| SHS |  |  |  |  |
| No | Ref. |  | Ref. |  |
| Yes | 1.48 [1.39–1.58] | <0.001 | 1.30 [1.11–1.52] | 0.001 |
| Family history of lung cancer |  |  |  |  |
| No | Ref. |  | Ref. |  |
| Yes | 0.99 [0.92–1.07] | 0.845 | 0.93 [0.78–1.11] | 0.432 |
| Occupational exposure to hazardous substances |  |  |  |  |
| No | Ref. |  | Ref. |  |
| Yes | 0.95 [0.82–1.10] | 0.457 | 1.11 [0.80–1.56] | 0.529 |
| Family history of other cancer |  |  |  |  |
| No | Ref. |  | Ref. |  |
| Yes | 0.95 [0.90–1.01] | 0.078 | 0.94 [0.79–1.12] | 0.480 |
| History of other cancers |  |  |  |  |
| No | Ref. |  | Ref. |  |
| Yes | 1.09 [0.97–1.22] | 0.147 | 1.23 [0.95–1.60] | 0.111 |
| COPD |  |  |  |  |
| No | Ref. |  | Ref. |  |
| Yes | 1.00 [0.90–1.12] | 0.991 | 1.20 [0.94–1.54] | 0.146 |
| Emphysema |  |  |  |  |
| No | Ref. |  | Ref. |  |
| Yes | 1.79 [1.50-2.14] | <0.001 | 2.61 [1.94–3.50] | <0.001 |
| Angina pectoris |  |  |  |  |
| No | Ref. |  | Ref. |  |
| Yes | 1.06 [0.89–1.26] | 0.532 | 1.48 [1.04–2.11] | 0.029 |
| Diabetes |  |  |  |  |
| No | Ref. |  | Ref. |  |
| Yes | 1.08 [0.97–1.22] | 0.167 | 1.26 [0.98–1.62] | 0.074 |
| Hypertension |  |  |  |  |
| No | Ref. |  | Ref. |  |
| Yes | 1.17 [1.09–1.25] | <0.001 | 1.50 [1.29–1.73] | <0.001 |
| Physical activity |  |  |  |  |
| Low | Ref. |  | Ref. |  |
| Medium | 0.76 [0.59–0.99] | 0.042 | 0.52 [0.33–0.83] | 0.005 |
| High | 0.70 [0.54–0.90] | 0.006 | 0.42 [0.27–0.65] | <0.001 |

BMI, body mass index; COPD, chronic obstructive pulmonary disease; SHS, second-hand smoking
