# Supplementary material for: Prevalence and risk factors of lung nodules in a non-smoking Chinese population: a prospective study of low-dose computed tomography screening
Source: BMC Pulm Med. 2026 Mar 4;26:165. doi: 10.1186/s12890-026-04108-2 (PMC13067505; doi:10.1186/s12890-026-04108-2)
Supplement: Supplementary file 3 — Additional file 3: Risk factors for different types of nodules by multivariate logistic regression analysis. [file 12890_2026_4108_MOESM3_ESM.docx]

**Additional file 3: Risk factors for different types of nodules by multivariate logistic regression analysis**

| **Characteristics** | **Lung noodles** | | **Clinically relevant nodules** | |
| --- | --- | --- | --- | --- |
|  | OR [95% CL] | *P* | OR [95% CL] | *P* |
| Sex |  |  |  |  |
| Male | Ref. |  | Ref. |  |
| Female | 1.12 [1.06–1.19] | <0.001 | 0.96 [0.84–1.10] | 0.577 |
| Age (years) range |  |  |  |  |
| <45 | Ref. |  | Ref. |  |
| 45–49 | 1.18 [1.10–1.28] | <0.001 | 1.57 [1.27–1.95] | <0.001 |
| 50–54 | 1.47 [1.36–1.60] | <0.001 | 1.95[1.58–2.41] | <0.001 |
| 55–59 | 1.67 [1.52–1.83] | <0.001 | 2.58 [2.08–3.21] | <0.001 |
| 60–64 | 2.01 [1.81–2.23] | <0.001 | 2.60 [2.04–3.31] | <0.001 |
| 65–69 | 2.498[2.18–2.83] | <0.001 | 3.71[2.85–4.83] | <0.001 |
| 70–74 | 3.10 [2.53–3.79] | <0.001 | 5.12 [3.66–7.15] | <0.001 |
| ≥75 | 2.60 [2.01–3.36] | <0.001 | 5.01 [3.30–7.59] | <0.001 |
| Educational level |  |  |  |  |
| Low | Ref. |  | Ref. |  |
| Medium | 0.92 [0.78–1.09] | 0.349 | 0.62 [0.46–0.82] | 0.001 |
| High | 0.89 [0.75–1.05] | 0.171 | 0.52 [0.39–0.69] | <0.001 |
| SHS |  |  |  |  |
| No | Ref. |  | Ref. |  |
| Yes | 1.59 [1.49–1.70] | <0.001 | 1.44 [1.22-1.69] | <0.001 |
| Emphysema |  |  |  |  |
| No | Ref. |  | Ref. |  |
| Yes | 1.49 [1.24–1.78] | <0.001 | 1.84 [1.36–2.49] | <0.001 |
| Angina pectoris |  |  |  |  |
| No | Ref. |  | Ref. |  |
| Yes | 0.89 [0.75–1.07] | 0.208 | 1.10 [0.77–1.58] | 0.608 |
| Hypertension |  |  |  |  |
| No | Ref. |  | Ref. |  |
| Yes | 0.97 [0.91–1.05] | 0.466 | 1.08 [0.93–1.26] | 0.327 |
| Physical activity |  |  |  |  |
| Low | Ref. |  | Ref. |  |
| Medium | 0.86 [0.66–1.12] | 0.272 | 0.67 [0.42–1.07] | 0.096 |
| High | 0.90 [0.70–1.17] | 0.448 | 0.67 [0.42–1.05] | 0.078 |

CL, confidence level; OR, odds ratio; SHS, second-hand smoking
